# Validation of the Antiproliferative Effects of Organic Extracts from the Green Husk of *Juglans regia* L. on PC-3 Human Prostate Cancer Cells by Assessment of Apoptosis-Related Genes

**DOI:** 10.1155/2012/103026

**Published:** 2012-02-06

**Authors:** Ali A. Alshatwi, Tarique N. Hasan, Gowhar Shafi, Naveed Ahmed Syed, Abdullah H. Al-Assaf, Mohammed S. Alamri, Abdrohman S. Al-Khalifa

**Affiliations:** Molecular Cancer Biology Research Laboratory (MCBRL), Department of Food Science and Nutrition, College of Food and Agricultural Sciences, King Saud University, P.O. Box 2460, Riyadh 11451, Saudi Arabia

## Abstract

With the increased use of plant-based cancer chemotherapy, exploring the antiproliferative effects of phytochemicals for anticancer drug design has gained considerable attention worldwide. This study was undertaken to investigate the effect of walnut green husk extracts on cell proliferation and to determine the possible molecular mechanism of extract-induced cell death by quantifying the expression of Bcl-2, Bax, caspases-3, and Tp53. PC-3 human prostate cancer cells. In this study, we found that green husk extracts suppressed proliferation and induced apoptosis in a dose- and time-dependent manner by modulating expression of apoptosis-related genes. This involved DNA fragmentation (determined by TUNEL assay) and significant changes in levels of mRNA and the expression of corresponding proteins. An increase in expressions of *Bax, caspase-3*, and *tp53* genes and their corresponding proteins was detected using real-time PCR and western blot analysis in PC-3 cells treated with the green husk organic extracts. In contrast, Bcl2 expression was downregulated after exposure to the extracts. Our data suggest the presence of bioactive compound(s) in walnut green husks that are capable of killing prostate carcinoma cells by inducing apoptosis and that the husks are a candidate source of anticancer drugs.

## 1. Introduction

Natural agents and herbal products can boost the actions and reduce the toxicity of conventional chemotherapeutic drugs [1]. The antineoplastic effects of natural agents and herbal products may affect cells by many different mechanisms, such as by preventing initiation and promotion of cancerogenesis or inducing apoptosis. Therefore, the identification of activators of apoptosis may help in providing more effective strategies for cancer therapy. The walnut, *Juglans regia *L., is an important deciduous tree species found principally in temperate areas across the world. It is cultivated throughout eastern Asia, southern Europe, northern Africa, the United States, western South America, and India. It is largely consumed as part of the diet, and different parts of plant are often used in local folk medicine. In addition, green walnuts, walnut shells, bark, green husks (epicarps), and leaves have been used in the cosmetic and pharmaceutical industries [2, 3].

Walnuts contain tocopherols and other nutrients that may protect against prostate cancer: 75 g of walnuts contain 0.52 mg *α*-tocopherols and 15.6 mg *γ*-tocopherols [4]. Walnuts also contain ellagic acid (590 *μ*g/g) [5], which has been shown to effectively induce apoptosis and inhibit angiogenesis [6, 7]. In addition, walnut kernels are a rich source of unsaturated fatty acids [8]; several recent studies have reported that consumption of 70–80 g walnuts per day reduces total and low-density lipoprotein cholesterol [9, 10].

With 543,000 new cases a year, prostate cancer is the third most common form of cancer in the world for men, according to the World Cancer Report 2003 [11]. Asian populations have the world's lowest incidence and mortality rates of prostate cancer; however, in the last two decades, these rates have risen rapidly in most Asian countries. At present, prostate cancer is one of the principal forms of male cancer in some Asian countries. In 2000, the age-adjusted incidence was over 10 per 100,000 men in Japan, Taiwan, Singapore, Malaysia, the Philippines, and Israel [12]. According to the 1998 cancer incidence report of the Gulf Cooperation Council countries, published by the Gulf Center for Cancer Registration, the highest age-standardized incidence rate of prostate cancer was in Oman and Kuwait (10.6/100,000), followed by Bahrain (10.3/100,000), Qatar (8.6/100,000), and the United Arab Emirates (7.1/100,000). The lowest was in Saudi Arabia, with an age-standardized incidence rate of 4.2/100,000 [13]; but according to a report by the Ministry of Health, Saudi Arabia, the age-standardized rate of prostate cancer incidence was 3.5/100,000 in a 1999-2000 survey [14]. Although the incidence of prostate cancer in Saudi Arabia is low compared with other Asian countries, the incidence appears to have risen over the last decade [15]. Hence, with increasing longevity, prostate cancer will become a growing problem for Saudi Arabia. Even after three decades of combating breast cancer, there is still no effective cure for this disease. However, according to some reports, the use of complementary alternative medicine has improved the condition of breast cancer patients [16].

Agents that suppress the proliferation of malignant cells by enhancing apoptosis may constitute a useful mechanistic approach to both cancer chemoprevention and chemotherapy. However, unfavorable side effects and resistance to many developed anticancer agents have been serious problems [17]. Thus, there is a growing interest in the use of plant-based compounds to develop safe, more effective therapeutic agents in cancer treatment [18]. Since the side effects of plant extracts are modest and well Hassan Magdyextracts from walnut leaves inhibit the growth of PC-3 cells through apoptosis and to ascertain the molecular mechanism involved in apoptosis.

## 2. Materials and Methods

### 2.1. Preparation of Three Organic Extracts with Increasing Polarity

Extractions were done as described earlier [19], with some modifications. Air-dried walnut green husks (WNGHs) were powdered in a milling machine. Extractions were carried out in a Soxhlet apparatus with three different solvents: methanol, chloroform, and n-hexane. Each organic phase was later dried under reduced pressure to obtain the residue. A rotary evaporator was used to obtain the respective lyophilized powder/paste. Extracts were weighed and stocks were prepared at a concentration of 100 mg/mL dimethyl sulfoxide.

### 2.2. Maintenance of PC-3 Cell Line

PC-3 prostate cancer cells were obtained from the National Center for Cell Science, Pune, India. The cell line was maintained and propagated in 90% Dulbecco's modified Eagle's medium containing 10% fetal bovine serum and 1% penicillin/streptomycin. The cells were cultured up to about 70% to 80% confluence as an adherent monolayer. Throughout the study, the cells were maintained at 37°C in a humidified atmosphere of 5% CO_2_. The cells were trypsinized for harvesting after attaining confluence. Only research-grade chemicals were used in this study.

### 2.3. Cell Viability Assay

According to the study design, cell viability was assayed by means of the trypan blue exclusion test [20] after thawing the cryopreserved vial, every time before seeding the cells. The overall viability was over 90%.

### 2.4. Cell Proliferation Assay and Determination of IC_50_

The Cell Titer Blue viability assay (Promega, Madison, WI, USA) was performed to assess the toxicity of different concentrations of WNGH organic extracts on PC-3 cells. The assay was performed according to the manufacturer's instructions. Briefly, PC-3 cells (2 × 10^4^ cells/well) were plated in 96-well plates and treated with 0, 5, 10, 20, 40, 80, and 160 *μ*g/mL of all three organic extracts (methanol, chloroform, and n-hexane) for 24 hours. Then, 40 *μ*L of the Cell Titer Blue solution was directly added to the wells and incubated at 37°C for 6 hours. The fluorescence was recorded by means of a 560-nm/590-nm (excitation/emission) filter set using a Bio-Tek microplate fluorescence reader (F Lx800), and the IC_50_ was calculated. Quadruplet samples were run for each concentration of the organic extracts in three independent experiments.

### 2.5. Cytochemical Quantification of Apoptosis by TUNEL Assay

The terminal deoxynucleotidyl transferase dUTP nick-end labeling (TUNEL) assay is an effective blend of molecular biology and morphological observation used largely for the quantification of apoptosis in cells [21]. In the present study, the TUNEL assay was conducted to investigate the occurrence of DNA fragmentation induced by organic extracts of RBJR. The DeadEnd TUNEL assay kit (Promega, Madison, WI, USA) was used for studying apoptosis in a time- and dose-dependent manner. The TUNEL assay was performed as described earlier [22]. Briefly, 2 × 10^4^ cells/well were cultured in six-well plates. The cells were treated with 50 *μ*g/mL and 100 *μ*g/mL of methanol, chloroform, and n-hexane separately for 24, 48, and 72 hours. The culture media were aspirated after the incubation period, and the cell layers were trypsinized. The trypsinized cells were reattached on 0.01% polylysine-coated slides, fixed with 4% methanol-free formaldehyde solution, and stained according to the DeadEnd fluorometric TUNEL system protocol. The stained cells were observed using a Carl-Zeiss (Axiovert) epifluorescence microscope using a triple-bandpass filter. Green-fluorescent (FITC stain) cells were regarded as TUNEL positive; cells with a red nucleus (PI stain) were regarded as TUNEL negative. To determine the cell count of TUNEL-positive cells, 1000 cells were counted at a magnification of ×100 in each experiment [23]. The apoptotic cell counts in the treated cells were normalized by the control count. Each experiment was repeated three times.

### 2.6. Quantification of mRNA Level of Apoptosis-Related Genes through Real-Time PCR 

PC-3 cells were cultured in six-well plates at a density of 1.5 × 10^5^. After 24 hours of adhesion, the culture media were aspirated off and the cells were treated with methanol, chloroform, and n-hexane extracts at a concentration of 50 *μ*g/mL medium. After 24 hours of incubation, the media were removed and the treated cells were washed with ice-cold phosphate-buffered saline (PBS). Fastlane Cell cDNA kit (QIAGEN, Germany) was used to prepare cDNA directly from the cultured cells, according to the manufacturer's instructions. The mRNA levels of *tp53*, *caspase-3*, *bax*, and *bcl-2 *as well as the reference gene, **β*-actin*, were assayed using gene-specific S YBR Green-based QuantiTect Primer assays (QIAGEN, Germany). Real-time PCR reactions and analyses were performed on an Applied Biosystems 7500 Fast (Foster City, CA, USA).

Quantitative real-time PCR was performed in a reaction volume of 25 *μ*L according to the manufacturer's instructions. Briefly, 12.5 *μ*L of the master mix, 2.5 *μ*L of primer assay (10x), and 10 *μ*L of template cDNA (100 *μ*g) were added to each well. After brief centrifugation, the PCR plate was subjected to 35 cycles of the following conditions: (i) PCR activation at 95°C for 5 minutes; (ii) denaturation at 95°C for 5 seconds; (iii) annealing/extension at 60°C for 10 seconds. All samples and controls were run in triplicate on an ABI 7500 Fast Real-Time PCR System. The quantitative RT-PCR data were analyzed by a comparative threshold (Ct) method, and the fold inductions of the samples were compared with the untreated samples. **β*-actin *was used as an internal reference gene to normalize the expression of the apoptotic genes. The Ct cycle was used to determine the expression level in control cells and PC-3 cells treated with different extracts for 24 hours. The gene-expression level was then calculated as described by Yuan et al. [24]. The results were expressed as the ratio of the reference gene to the target gene using the following formula: ΔCt = Ct (apoptotic genes) − Ct (**β*-actin*). To determine the relative expression levels, the following formula was used: ΔΔCt = ΔCt (treated) − ΔCt (control). Thus, the expression levels were presented as *n*-fold differences relative to the calibrator. The value was used to plot the expression of apoptotic genes using the expression of 2^−ΔΔCt^.

### 2.7. Apoptosis-Related Protein Expression Analysis by Immunoblotting

Immunoblotting analysis of apoptosis-related proteins was performed as we described earlier [19, 23], with a slight modification. Briefly, 2 × 10^5^ cells/well were cultured in six-well plates. The cells were then treated with methanol, chloroform, and n-hexane at a concentration of 50 *μ*g/mL for 24 hours. After treatment, the cells were collected and washed twice with cold PBS. The cells were then lysed in lysis buffer (50 mM Tris-HCl, pH 7.5, 150 mM NaCl, 1% Nonidet P-40, 2 mM EDTA, 1 mM EGTA, 1 mM NaVO3, 10 mM NaF, 1 mM DTT, 1 mM PMSF, 25 *μ*g/mL aprotinin, and 25 *μ*g/mL leupeptin) and kept on ice for 30 min. The lysates were then centrifuged at 12,000 ×g at 4°C for 20 min; the supernatants were stored at –70°C until use. The protein concentration was determined using the Bradford method. Aliquots of the lysates (30 *μ*g protein) were separated by 12% SDS-PAGE and transferred to a nitrocellulose membrane using transfer buffer (192 mM glycine, 25 mM Tris-HC l, pH 8.8, and 20% methanol (v/v)). After blocking with 5% nonfat dried milk, the membrane was incubated for 2 hours with primary antibodies, followed by 30 min with secondary antibodies in milk-containing tris-buffered saline (TBS) and 0.5% Tween. Anti-human p53, caspase-3, Bcl-2, Bax, and *β*-actin antibodies were used at a 1 : 1000 dilution as the primary antibodies, while horseradish peroxidase-conjugated horse anti-rabbit IgG (Sigma Chemicals, USA) was used at a 1 : 5000 dilution as the secondary antibody. The membrane was then exposed, and protein bands were detected using enhanced chemiluminescence. All chemicals used during this study were of research grade.

### 2.8. Statistical Analysis

All data were analyzed statistically using PASW 18 software and represented as means ± SEM. A *t* test was performed, and a *P *value ≤0.05 was considered significant. 

## 3. Results

### 3.1. WNGH Extract-Induced Clonogenic Inhibition of PC-3 Cells

**Figure 1:**
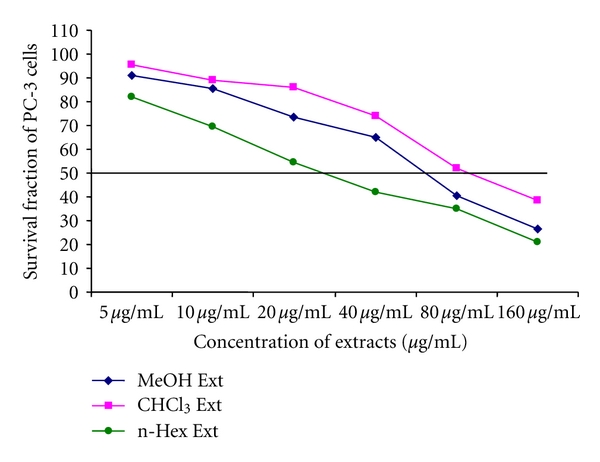
Survival fractions. PC-3 cells treated with methanol, chloroform, and n-hexane extracts of WNGH, showing the IC_50_ against PC-3 cells.

The methanol (IC_50_ 66.72 *μ*g/mL), n-hexane (IC_50_ 27.29 *μ*g/mL), and chloroform (IC_50_ 91.14 *μ*g/mL) extracts significantly inhibited the growth of PC-3 cells (Figure 1). Further, these three extracts produced significant cytotoxicity to PC-3 cells. The IC_50_ values clearly indicated that n-hexane had a higher growth-inhibiting effect on PC-3 cells than n-hexane and methanol.

### 3.2. Quantitative Assessment of Apoptosis by TUNEL Assay

**Figure 2:**
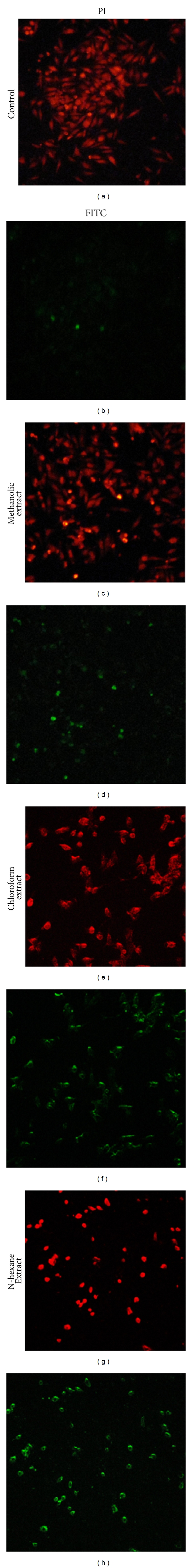
Morphological observation with propidium iodide/FITC double staining by fluorescence microscopy (×100). Cells were cultured in six-well plates. Control cells were untreated (a, b). Test cells were subjected to treatment with IC_50_ of methanol (c, d), chloroform (e, f), and n-hexane extracts (g, h) separately for three different incubation periods (24, 48, and 72 hours). TUNEL assay was performed and TUNEL-positive cells (green fluorescent)—the apoptotic cells—were scored out of 1000 cells counted/treated culture of PC-3 cells. Representative images from three independent experiments.

**Figure 3:**
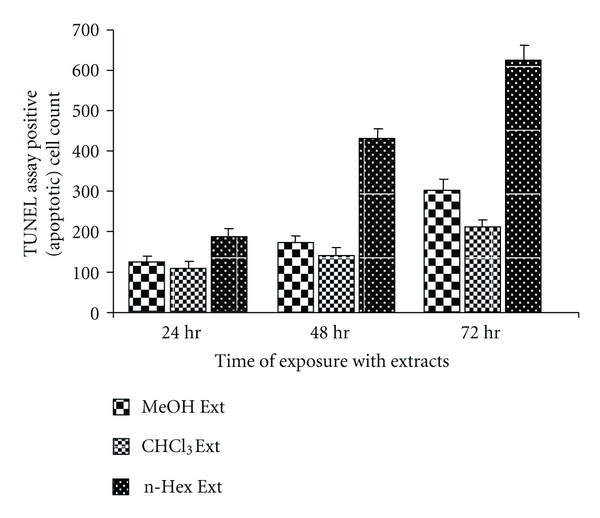
Score of TUNEL-positive cells. 1000 cells were counted after each incubation period after staining with propidium iodide and FITC; TUNEL-positive (green fluorescent) cells were scored and normalized with controls (untreated cells). Data shown are means ± SEM for three independent experiments. MeOH Ext, methanol extract; CH Ext, chloroform extract; n-Hex Ext, n-hexane extract.

To determine the apoptotic cell count, PC-3 cells were treated with respective IC_50_ concentration of WNGH extracts. The PC-3 cells treated with the extracts showed active apoptosis in a time- and dose-dependent manner. The increase in TUNEL-positive apoptotic cell count (out of 1000 cells counted) over the untreated population for 24-, 48-, and 72-hour treatment was, respectively, 124 ± 11, 170 ± 16, and 301 ± 24 for methanol; 107 ± 14, 140 ± 19, and 210 ± 15 for n-hexane; and 184 ± 20, 428 ± 25, and  622 ± 43  for chloroform (Figure 3). Apoptosis was determined by microscopy-based TUNEL examination (Figure 2).

### 3.3. Determination of Apoptosis-Related Genes by Real-Time PCR

To investigate the molecular mechanism of WNGH extract-induced apoptosis in PC-3 cells, the expression levels of several apoptosis-related genes were examined. Bcl-2, Bax, and p53 are three major proteins that are generally involved in apoptosis. It is not known whether LE extract induces or inhibits the expression of these genes. The relative quantification of *tp53*, *caspase-3*, *bax*, and *bcl-2 *mRNA levels was performed.

**Figure 4:**
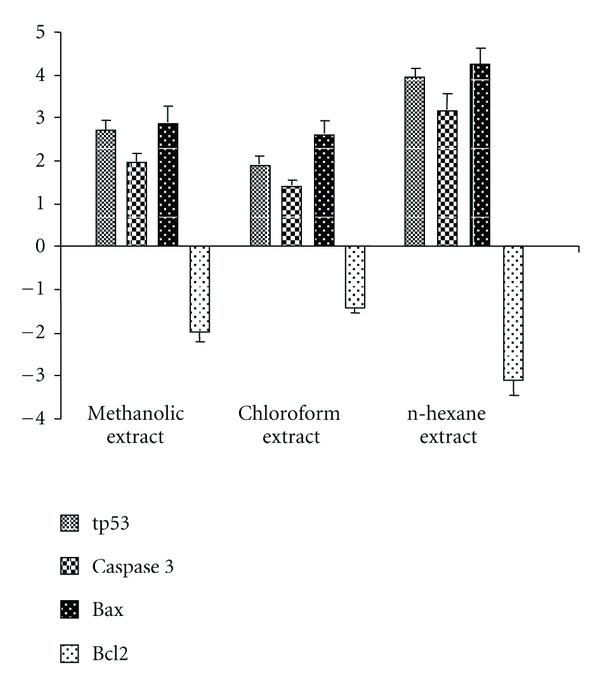
Fold change in expression of mRNA of apoptosis-related genes against controls over 24 hours. Real-time polymerase chain reaction analysis of apoptosis-related genes showed that *tp53*, *caspase-3*, and *bax *were up-regulated in cells treated with all three extracts, but bcl-2 was downregulated. Data represented are means ± SEM of three independent experiments.

Figure 4 summarizes the gene-expression changes of *tp53*, *caspase-3*, *bax*, and *bcl-2. *WNGH extract treatment caused a several-fold increase in the number of transcripts of *tp53*, *caspase-3*, and *bax*. The expression levels of these genes in PC-3 cells treated with WNGH methanol extract for 24 hours increased by 2.73 ± 0.19, 1.96 ± 0.16, and 2.87 ± 0.18, respectively. Compared with levels in untreated control cells, the increase in the mRNA level for *tp53*, *caspase-3*, and *bax *was, respectively, 1.87 ± 0.11, 1.14 ± 0.11, and 2.6 ± 0.29 for cells treated with chloroform and 3.94 ± 0.24, 3.18 ± 0.38, and 4.26 ± 0.39 for cells treated with n-hexane. A decrease in bcl2 expression was observed with all three treatments. The lowest value of expression was found in n-hexane-treated PC-3 cells (−3.11 ± 0.39); *bax *expression significantly increased in WNGH extract-treated cells (Figure 4), which indicates that the WNGH-extract treatment induced apoptosis by shifting the Bax : Bcl-2 ratio in favor of apoptosis.

### 3.4. Determination of Apoptosis-Related Proteins by Protein Immunoblotting

**Figure 5:**
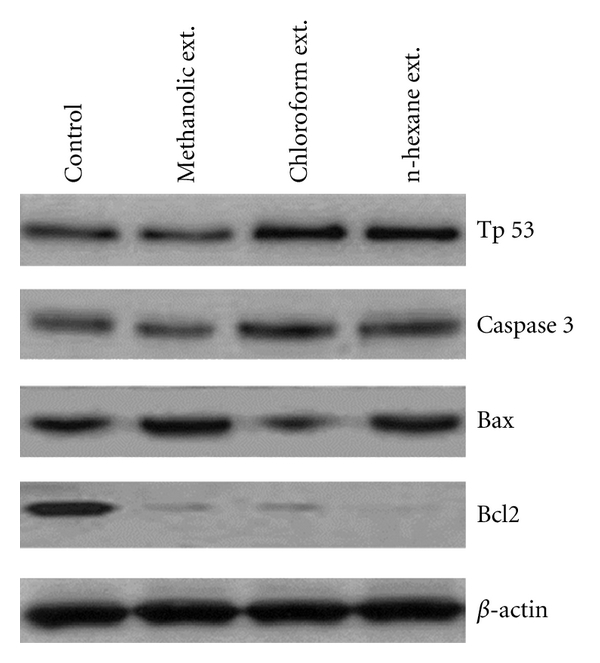
Protein immunoblotting/western blotting of apoptosis-related proteins. Protein-expression study showed upregulation of Tp53, Caspase-3, and Bax but downregulation of Bcl-2.

Protein immunoblotting results indicated that compared with controls, the expression of Tp53, Caspase-3, and Bax proteins was higher in n-hexane-treated PC-3 cells followed, respectively, by those treated with methanol and chloroform. However, Bcl-2 was down-regulated in the treatment of PC-3 cells with all three extracts (Figure 5).

## 4. Discussion

Agents suppressing the proliferation of malignant cells by enhancing apoptosis may constitute a useful mechanistic approach to both cancer chemoprevention and chemotherapy. Many anticancer agents have been developed, but unfavorable side effects and resistance are serious problems. There is, therefore need for more effective anticancer agents. Over the past few decades, screening medicinal plants and their parts for anticancer properties has developed as a major field of interest in medicine and chemistry. In our earlier study, we demonstrated that organic extracts of *Juglans regia* L. root bark are able to inhibit cell proliferation and induce apoptosis in MDA-MB 231 breast cancer cells [19]. In the present study, the anticancer property of WNGHs was evaluated in PC-3 cells. WNGHs were extracted using different organic solvents (methanol, chloroform, and n-hexane).

The results demonstrate that WNGHs suppressed the proliferation of PC-3 cells, and apoptosis was induced in a dose- and time-dependent manner. The IC_50_ values clearly indicated that the n-hexane extract had a much more potent effect on PC-3 cells than the chloroform and methanol extracts (Figure 2). Walnuts contain various compounds—omega-3 fatty acids, phytosterols, polyphenols, carotenoids, and melatonin—that may inhibit cancer growth and decrease tumor cell proliferation [25]. Juglone (5-hydroxy-1,4-naphthoquinone) is a chemical present in walnut trees that can be toxic at various levels [26], and it has antitumor activities against melanoma cells. Induction of oxidative stress, cell membrane damage, and a clastogenic action contribute to the cytotoxic effect of juglone [27]. Using transmission electron microscopy, Ji et al. have also reported the typical morphological changes of apoptosis, such as chromatin condensation, margination against the nuclear envelope and the formation of apoptotic bodies, after juglone treatment [28].

To understand the potential antitumor mechanisms of WNGH, the expression of pro- and antiapoptotic genes was investigated in the present study. We found decreased expression of *Bcl-2 *mRNA and protein, but increased expression of *Bax, caspase3*, and *p53 *mRNA and protein; therefore, the *Bax* : *Bcl-2 *ratio was elevated. In addition, the expression of p53, caspase-3, and Bax mRNA and proteins was highest in n-hexane-treated PC-3 cells followed, respectively, by those treated with methanol and chloroform compared with controls. However, Bcl-2 mRNA and protein were downregulated in PC-3 cells treated with all three extracts compared with controls. The data presented in this study suggest that WNGH-induced apoptosis is mediated by the death receptor and mitochondrial apoptotic pathways, as demonstrated by increased expression levels of caspase-3 after WNGH treatment.

These pro- and antiapoptotic proteins are the principal regulators of the intrinsic pathway of apoptosis [29]. Previous reports have shown that the ratio of Bax to Bcl-2 determines, in part, the susceptibility of cells to death signals [30]. In the present study, Bcl-2 expression was significantly inhibited, whereas the expression of Bax, Tp53, and caspase-3 markedly increased in all extract treatments.

WNGH extract may induce apoptosis through p53, through the downregulation of *Bcl-2 *and *Mdm2*, and through upregulation of *Bax*. The Bcl-2 family is one of the most important classes of regulators involved in the intrinsic pathway. The functions of *Bax *and *Bcl-2 *are known to be upstream of caspases that regulate apoptosis promoted by different stimuli [31].

The possible mechanism by which *p53 *regulates apoptosis involves activating the mitochondria-regulated death pathway by elevating gene expression of proapoptosis genes in the Bcl-2 family and suppressing the expression of antiapoptotic genes [32, 33]. *p53 *protein interacts with *Bcl-2 *to enhance Bax-promoted outer-mitochondrial membrane permeabilization, and *p53 *is a direct transcriptional activator of the *Bax *gene [34, 35].

Increased expression of *p53 *induces an increase in the *Bax* : *Bcl-2 *ratio, resulting in *cytochrome-c, caspase *activation, and, ultimately, apoptosis. One possible mechanism by which *Bax *may function in the *p53*-mediated cell-death pathway is through the activation of caspases: *p53*-mediated activation of *caspase*-3 is dependent on *Bax *[36]. *Bax *is required for *caspase *activation after the potassium withdrawal-enhanced cell death of cerebellar granule neurons [37]. Furthermore, Bax overexpression can promote caspase activation in neuronal cells [38, 39]. Our data suggest that WNGH extract may induce cell apoptosis mediated by *p53*. Apoptosis is also the result of death receptor-dependent (extrinsic) or death receptor-independent (intrinsic or mitochondrial) mechanisms [40].

By inducing the release of mitochondrial *cytochrome c, p53 *may be able to activate effector caspases, including *caspase*-3. WNGH extract may induce apoptosis mediated by *p53 *through the downregulation of *Bcl*-2 and upregulation of *Bax*. WNGH extract has shown antitumor effects on breast cancer cells, and the present results suggest that WNGH extracts can induce *p53*-mediated apoptosis via modulation of the *Bax* : *Bcl-2 *ratio. Protein-expression data are needed to confirm the changes observed at the mRNA level.

Based on the bioactivity of these compounds from WNGHs, their identification and determination will play an important role in the safe, effective use of WNGHs for pharmaceutical purpose. Therefore, it is necessary to develop an analytical method to identify and determine these compounds.

According to present knowledge, WNGHs contain a battery of bioactive compounds of different chemical types, including flavanoids, naphthoquinones, diarylheptanoids, triterpenes, phenol acid, sterol, and lipid substances. The total phenol content in WNGH extracts may differ according to the solvent used for extraction. However, all three organic extracts used in the present study were able to induce apoptosis owing to their respective bioactive compounds; however, the higher toxic activity of n-hexane extract suggests the presence of a greater amount of bioactive compounds. Further study is required to characterize WNGH organic extracts and to identify the molecules responsible for this bioactivity. On the other hand, the potential observed with WNGH organic extracts may lead to the valorization of a by-product that currently has scarce use.

In conclusion, the present study demonstrated that the WNGH extract inhibited cancer cell proliferation via the induction of apoptosis. WNGH extract-induced MCF-7 cell death was shown to be due to apoptosis, as demonstrated by the induction of caspase-3 activity and the observation of cells containing fragmented nuclei and DNA.

As far we know, we have demonstrated for the first time that the apoptotic response is associated with upregulation of *Bax*, downregulation of *Bcl-2*, and caspase activation. Therefore, we suggest that WNGH extract may be a good source of promising molecules in cancer chemoprevention or chemotherapy. These results indicate that the WNGH extract has an anticancer activity *in vitro*. Further studies are necessary to determine the molecular mechanisms of the active components and to evaluate the potential *in vivo* anticancer activity of the extract.
